# Supplementary material for: Causal Inference of Central Nervous System-Regulated Hormones in COVID-19: A Bidirectional Two-Sample Mendelian Randomization Study
Source: J Clin Med. 2023 Feb 20;12(4):1681. doi: 10.3390/jcm12041681 (PMC9961400; doi:10.3390/jcm12041681)
Supplement: Supplementary file 1 [file jcm-12-01681-s001.zip › jcm-2181545-supplementary.pdf]

**Supplementary Information**

**Table S1.** Information of data source and candidate genetic instruments.

(a) CNS-regulated Hormones. We were not able to find any LD proxy SNPs. The sample size was the maximum number indicated in the published source. All hormone traits except TSH was obtained from IEU open GWAS project (https://gwas.mrcieu.ac.uk/) and GWAS ID was the ID number of these exposure on the website. TSH was obtained from study of Porcu E. et al.

| Exposure | Sample Size | Number of SNPs | | | Variable adjusted | GWAS ID/Data source |
|---|---|---|---|---|---|---|
| | | Severity | Hospitalization | Susceptibility | | |
| IGF-1 | 468,343 | 613 | 604 | 611 | age, age$^2$, sex, age sex, age$^2$, sex, PCs | ukb-d-30770_irnt |
| Estradiol | 427,024 | 1 | 1 | 1 | | ukb-d-30800_irnt |
| Testosterone | 77,700 | 130 | 130 | 129 | | ukb-d-30850_irnt |
| DHEA-S | 50,000 | 2 | 2 | 2 | age, sex, batch effects | met-a-478 |
| TSH | 26,420 | 12 | 13 | 12 | age, age$^2$, sex | A meta-analysis[1] |
| TRH | 26,420 | 1 | 1 | 1 | age, sex, time duration, PCs | prot-a-3102t |
| LH | 50,000 | 1 | 1 | 1 | | prot-a-529 |
| FSH | 1,000 | 1 | 1 | 1 | age, sex, body mass index | prot-c-3032_11_2 |

Note: PC is the abbreviation of principal component. [1]Porcu, Eleonora, et al. "A meta-analysis of thyroid-related traits reveals novel loci and gender-specific differences in the regulation of thyroid function." PLoS genetics 9.2 (2013): e1003266.

(b). COVID-19. The candidate genetic instruments were used in reverse direction of MR analysis. There were no matched SNPs with TSH.

| Exposure | Sample size | Number of SNPs | | | | | | | |
|---|---|---|---|---|---|---|---|---|---|
| | | Total | DHEA-S | Estradiol | LH | Testosterone | FSH | IGF-1 | TRH |
| Very severe respiratory syndromes | 714,592 | 9,856,861 | 6 | 8 | 8 | 8 | 4 | 8 | 8 |
| Hospitalization | 1,828,485 | 8,152,576 | 4 | 5 | 5 | 5 | 3 | 5 | 5 |
| Susceptibility | 2,298,674 | 8,738,878 | 6 | 7 | 7 | 7 | 3 | 7 | 7 |

**Table S2**. Estimated variance explained by SNPs and F statistics.

| | **Very severe respiratory syndromes** | | **Hospitalization** | | **Susceptibility** | |
|---|---|---|---|---|---|---|
| Exposure | Estimated variance explained (%) | F statistics | Estimated variance explained (%) | F statistics | Estimated variance explained (%) | F statistics |
| DHEA-S | 1.44 | 53.75 | 1.44 | 53.75 | 1.44 | 53.75 |
| Estradiol | 0.07 | 37.66 | 0.07 | 37.66 | 0.07 | 37.66 |
| LH | 6.30 | 221.77 | 6.30 | 221.77 | 6.30 | 221.77 |
| Testosterone | 3.33 | 82.65 | 3.31 | 82.06 | 3.27 | 81.14 |
| FSH | 17.88 | 215.92 | 17.88 | 215.92 | 17.88 | 215.92 |
| IGF-1 | 13.72 | 88.67 | 13.60 | 87.78 | 13.68 | 89.67 |
| TSH | 2.95 | 66.88 | 2.95 | 61.73 | 2.95 | 66.88 |
| TRH | 1.01 | 33.61 | 1.01 | 33.61 | 1.01 | 33.61 |

Note: Estimated variance explained (%) was calculated as: $R^2$ = (reported GWAS effect)$^2$ × 2 × minor allele frequency × (1 – minor allele frequency) / variance of trait. F statistics per variant was calculated as: (Sample size – 2) × $R^2$ / (1 – $R^2$).

**Table S3.** MR analyses of CNS-regulated hormone SNPs and COVID-19 very severe respiratory syndromes, hospitalization, and susceptibility.

| | | Very severe respiratory syndromes | | Hospitalization | | Susceptibility | |
|---|---|---|---|---|---|---|---|
| **Exposure** | **Method** | **OR (95% CI)** | ***p*** | **OR (95% CI)** | ***p*** | **OR (95% CI)** | ***p*** |
| DHEA-S | IVW | 4.21 (1.41 - 12.59) | 0.010 | 2.31 (1.13 - 4.72) | 0.021 | 1.09 (0.76 - 1.56) | 0.655 |
| Estradiol | Wald Ratio | 1.73 (0.32 - 9.37) | 0.524 | 1.20 (0.64 - 2.24) | 0.571 | 1.15 (0.34 - 3.83) | 0.822 |
| LH | Wald Ratio | 0.83 (0.71 - 0.96) | 0.013 | 0.91 (0.82 - 1.00) | 0.061 | 0.96 (0.92 - 1.00) | 0.115 |
| Testosterone* | IVW | 0.94 (0.60 - 1.47) | 0.794 | 1.10 (0.84 - 1.45) | 0.482 | 0.92 (0.81 - 1.05) | 0.237 |
| | MR-Egger | 1.54 (0.71 - 3.35) | 0.275 | 1.37 (0.85 - 2.22) | 0.205 | 1.16 (0.91 - 1.47) | 0.243 |
| | WM | 1.46 (0.75 - 2.84) | 0.266 | 1.30 (0.85 - 1.99) | 0.219 | 1.04 (0.85 - 1.28) | 0.691 |
| FSH | Wald Ratio | 0.83 (0.59 - 1.16) | 0.268 | 0.87 (0.69 - 1.09) | 0.221 | 0.92 (0.82 - 1.04) | 0.174 |
| IGF-1* | IVW | 0.97 (0.86 - 1.10) | 0.652 | 1.00 (0.92 - 1.09) | 0.987 | 0.99 (0.95 - 1.03) | 0.580 |
| | MR-Egger | 0.95 (0.73 - 1.24) | 0.731 | 1.02 (0.86 - 1.21) | 0.828 | 1.01 (0.93 - 1.10) | 0.758 |
| | WM | 0.94 (0.76 - 1.15) | 0.524 | 1.00 (0.87 - 1.15) | 0.972 | 0.98 (0.92 - 1.05) | 0.594 |
| TSH | IVW | 0.96 (0.77 - 1.20) | 0.729 | 0.93 (0.79 - 1.09) | 0.368 | 0.99 (0.90 - 1.08) | 0.777 |
| | MR-Egger | 1.66 (0.80 - 3.34) | 0.199 | 1.20 (0.73 - 1.98) | 0.479 | 1.11 (0.84 - 1.47) | 0.475 |
| | WM | 0.86 (0.63 - 1.17) | 0.327 | 1.00 (0.81 - 1.23) | 0.979 | 1.05 (0.94 - 1.16) | 0.362 |
| TRH | Wald Ratio | 1.36 (0.84 - 2.21) | 0.212 | 1.08 (0.81 - 1.44) | 0.589 | 1.09 (0.95 - 1.25) | 0.223 |

Note: * indicated the estimator was outlier corrected by the MR-PRESSO method. The associations between IGF-1 and hospitalization and susceptibility were outlier corrected. The association between testosterone and susceptibility was outlier corrected.

**Table S4.** Bidirectional MR Analysis using COVID-19 very severe respiratory syndromes, hospitalization, and susceptibility as exposures.

| | | Very severe respiratory syndromes | | Hospitalization | | Susceptibility | |
|---|---|---|---|---|---|---|---|
| Outcome | **Method** | **OR (95% CI)** | ***p*** | **OR (95% CI)** | ***p*** | **OR (95% CI)** | ***p*** |
| DHEA-S | IVW | 1.00 (0.98 - 1.02) | 0.771 | 1.01 (0.98 - 1.03) | 0.658 | 1.01 (0.94 - 1.08) | 0.419 |
| | MR Egger | 0.99 (0.93 - 1.05) | 0.704 | 1.00 (0.94 - 1.06) | 0.899 | 0.97 (0.80 - 1.18) | 0.263 |
| | WM | 1.00 (0.98 - 1.03) | 0.732 | 1.00 (0.97 - 1.03) | 0.874 | 1.01 (0.93 - 1.10) | 0.985 |
| Estradiol | IVW | 1.01 (0.99 - 1.03) | 0.335 | 1.01 (0.97 - 1.05) | 0.669 | 0.98 (0.93 - 1.03) | 0.458 |
| | MR Egger | 1.02 (0.97 - 1.06) | 0.582 | 1.02 (0.93 - 1.11) | 0.771 | 1.01 (0.88 - 1.16) | 0.919 |
| | WM | 1.01 (0.99 - 1.03) | 0.443 | 1.01 (0.98 - 1.04) | 0.603 | 0.98 (0.93 - 1.05) | 0.612 |
| LH | IVW | 0.97 (0.88 - 1.05) | 0.409 | 0.98 (0.85 - 1.12) | 0.737 | 1.08 (0.85 - 1.54) | 0.526 |
| | MR Egger | 0.95 (0.77 - 1.18) | 0.677 | 0.97 (0.72 - 1.29) | 0.828 | 0.79 (0.38 - 1.39) | 0.524 |
| | WM | 0.97 (0.87 - 1.09) | 0.618 | 0.98 (0.84 - 1.15) | 0.834 | 1.09 (0.79 - 1.63) | 0.596 |
| Testosterone * | IVW | 1.00 (0.98 - 1.01) | 0.338 | 1.00 (0.98 - 1.01) | 0.748 | 1.05 (1.03 - 1.06) | <0.001 |
| | MR Egger | 0.98 (0.95 - 1.11) | 0.355 | 0.98 (0.94 - 1.01) | 0.296 | 0.97 (0.92 - 1.04) | 0.440 |
| | WM | 0.99 (0.98 - 1.00) | 0.077 | 0.99 (0.98 - 1.00) | 0.027 | 1.04 (1.01 - 1.06) | 0.002 |
| FSH | IVW | 1.06 (0.90 - 1.25) | 0.498 | 1.04 (0.82 - 1.32) | 0.728 | 0.85 (0.53 - 1.38) | 0.517 |
| | MR Egger | 0.98 (0.68 - 1.42) | 0.940 | 0.95 (0.56 - 1.60) | 0.873 | 1.13 (0.36 - 3.57) | 0.836 |
| | WM | 1.02 (0.88 - 1.19) | 0.839 | 1.00 (0.82 - 1.22) | 0.978 | 0.86 (0.50 - 1.48) | 0.584 |
| IGF-1 * | IVW | 1.01 (1.00 - 1.02) | 0.040 | 1.01 (0.99 - 1.02) | 0.339 | 0.99 (0.94 - 1.03) | 0.793 |
| | MR Egger | 0.96 (0.86 - 1.06) | 0.431 | 0.99 (0.96 - 1.02) | 0.498 | 0.96 (0.83 - 1.09) | 0.530 |
| | WM | 1.01 (1.00 - 1.02) | 0.271 | 1.00 (0.99 - 1.02) | 0.716 | 0.99 (0.95 - 1.02) | 0.461 |
| TSH | / | / | / | / | / | / | / |
| TRH | IVW | 1.06 (0.97 - 1.15) | 0.203 | 1.08 (0.94 - 1.24) | 0.290 | 0.93 (0.73 - 1.19) | 0.559 |
| | MR Egger | 1.10 (0.88 - 1.37) | 0.448 | 1.03 (0.73 - 1.44) | 0.890 | 0.82 (0.40 - 1.67) | 0.608 |
| | WM | 1.04 (0.93 - 1.16) | 0.523 | 1.04 (0.88 - 1.23) | 0.642 | 1.00 (0.73 - 1.35) | 0.974 |

Note: * indicated the estimator was outlier corrected by MR-PRESSO. The associations between IGF-1 and hospitalization and susceptibility were outlier corrected. The association between testosterone and susceptibility was outlier corrected.

**Table S5**. MVMR analysis using COVID-19 as exposures

| Exposure | IGF-1 | | Testosterone | |
|---|---|---|---|---|
| | OR (95% CI) | *p* | OR (95% CI) | *p* |
| Very severe respiratory syndromes | 1.05 ( 0.95 - 1.17) | 0.359 | 1.11 ( 1.03 - 1.19) | 0.003 |
| Hospitalization | 0.94 ( 0.86 - 1.02) | 0.150 | 0.89 ( 0.84 - 0.94) | <0.001 |
| Susceptibility | 1.00 ( 0.74 - 1.36) | 0.990 | 0.94 ( 0.77 - 1.15) | 0.564 |

**Table S6.** Cochran Test of heterogeneity, Measurement Error test, and MRPRESSO Test for horizontal pleiotropy of forward direction MR analysis

(a) Very severe respiratory syndromes as outcome

| | **MR-Egger Intercept test** | **Cochran test of Heterogeneity** | **Measurement Error Test** | **MRPRESSO Test** | | |
|---|---|---|---|---|---|---|
| | *p* | *p* | $I^2_{GX}$ | Globe Test *p* | Corrected IVW OR | *p* |
| IGF-1 | 0.885 | 0.001 | 0.936 | 0.001 | / | / |
| Testosterone | 0.129 | <0.001 | 0.946 | <0.001 | / | / |
| TSH | 0.098 | 0.820 | 0.808 | 0.579 | / | / |

(b) Hospitalization as outcome

| | **MR-Egger Intercept test** | **Cochran test of Heterogeneity** | **Measurement Error Test** | **MRPRESSO Test** | | |
|---|---|---|---|---|---|---|
| | *p* | *p* | $I^2_{GX}$ | Globe Test *p* | Corrected IVW OR | *p* |
| IGF-1 | 0.801 | < 0.001 | 0.931 | < 0.001 | 1.00 | 0.987 |
| Testosterone | 0.288 | 0.028 | 0.941 | 0.026 | / | / |
| TSH | 0.280 | 0.313 | 0.844 | 0.323 | / | / |

(c) Susceptibility as outcome

| | **MR-Egger Intercept test** | **Cochran test of Heterogeneity** | **Measurement Error Test** | **MRPRESSO Test** | | |
|---|---|---|---|---|---|---|
| | *p* | *p* | $I^2_{GX}$ | Globe Test *p* | Corrected IVW OR | *p* |
| IGF-1 | 0.547 | 0.007 | 0.928 | 0.007 | 0.99 | 0.580 |
| Testosterone | 0.057 | 0.050 | 0.940 | 0.037 | 0.96 | 0.237 |
| TSH | 0.379 | 0.110 | 0.858 | 0.122 | / | / |

**Table S7**. Cochran Test of heterogeneity, Measurement Error test and MRPRESSO Test for horizontal pleiotropy of bidirectional MR analysis

(a) Very severe respiratory syndrome as exposure

| | **MR-Egger Intercept test** | **Cochran test of Heterogeneity** | **Measurement Error Test** | **MRPRESSO Test** | | |
|---|---|---|---|---|---|---|
| | *p* | *p* | $I^2_{GX}$ | Globe Test *p* | Corrected IVW OR | *p* |
| IGF-1 | 0.204 | <0.001 | 0.847 | <0.001 | 1.01 | 0.040 |
| Estradiol | 0.820 | 0.264 | 0.846 | 0.437 | / | / |
| Testosterone | 0.392 | <0.001 | 0.847 | <0.001 | 1.00 | 0.388 |
| DHEA-S | 0.550 | 0.091 | 0.941 | 0.206 | / | / |
| TSH | / | / | / | / | / | / |
| TRH | 0.725 | 0.368 | 0.828 | 0.525 | / | / |
| LH | 0.907 | 0.770 | 0.838 | 0.848 | / | / |
| FSH | 0.646 | 0.117 | 0.826 | 0.354 | / | / |

(b) Hospitalization as exposure

| | **MR-Egger Intercept test** | **Cochran test of Heterogeneity** | **Measurement Error Test** | **MRPRESSO Test** | | |
|---|---|---|---|---|---|---|
| | *p* | *p* | $I^2_{GX}$ | Globe Test *p* | Corrected IVW OR | *p* |
| IGF-1 | 0.166 | 0.6615 | 0.913 | 0.525 | / | / |
| Estradiol | 0.872 | 0.069 | 0.914 | 0.266 | / | / |
| Testosterone | 0.212 | 0.0035 | 0.914 | 0.022 | 1.00 | 0.747 |
| DHEA-S | 0.679 | 0.3039 | 0.949 | 0.591 | / | / |
| TSH | / | / | / | / | / | / |
| TRH | 0.743 | 0.2526 | 0.913 | 0.509 | / | / |
| LH | 0.929 | 0.4401 | 0.913 | 0.687 | / | / |
| FSH | 0.660 | 0.1045 | 0.960 | / | / | / |

(c) Susceptibility as exposure

| | **MR-Egger Intercept test** | **Cochran test of Heterogeneity** | **Measurement Error Test** | **MRPRESSO Test** | | |
|---|---|---|---|---|---|---|
| | *p* | *p* | $I^2_{GX}$ | Globe Test *p* | Corrected IVW OR | *p* |
| IGF-1 | 0.669 | <0.001 | 0.661 | 0.003 | 0.99 | 0.793 |
| Estradiol | 0.694 | 0.504 | 0.655 | 0.643 | / | / |
| Testosterone | 0.077 | 0.057 | 0.661 | 0.023 | 1.05 | <0.001 |
| DHEA - S | 0.681 | 0.863 | 0.903 | 0.916 | / | / |
| TSH | / | / | / | / | / | / |
| TRH | 0.715 | 0.650 | 0.644 | 0.797 | / | / |
| LH | 0.361 | 0.394 | 0.644 | 0.466 | / | / |
| FSH | 0.597 | 0.786 | 0.755 | / | / | / |

**Table S8**. MR analysis of associations of sex stratified hormones with COVID-19 infections

(a) Very severe respiratory syndromes

| Sex | Exposure | Significant | Matched | Used | OR (95% CI) | *p* |
|---|---|---|---|---|---|---|
| **Female** | Estrogen | 20 | 10 | 1 | 1.51 (0.43 - 5.33) | 0.524 |
| | Testosterone | 7909 | 5075 | 96 | 1.16 (0.93 - 1.45) | 0.191 |
| | IGF-1 | 396550 | 24046 | 268 | 0.96 (0.83 - 1.11) | 0.553 |
| | TSH | 11 | 8 | 8 | 0.87 (0.67 - 1.13) | 0.296 |
| **Male** | Estrogen | 236 | 2 | 1 | 0.51 (0.19 - 1.32) | 0.163 |
| | Testosterone | 27658 | 14864 | 157 | 0.92 (0.79 - 1.08) | 0.297 |
| | IGF-1 | 41186 | 25510 | 270 | 1.03 (0.91 - 1.18) | 0.626 |
| | TSH | 13 | 8 | 8 | 0.86 (0.69 - 1.06) | 0.155 |

(b) Hospitalization

| Sex | Exposure | Significant | Matched | Used | OR (95% CI) | *p* |
|---|---|---|---|---|---|---|
| **Female** | Estrogen | 20 | 11 | 1 | 1.11 (0.45 - 2.73) | 0.822 |
| | Testosterone | 7909 | 4940 | 94 | 1.11 (0.97 - 1.28) | 0.132 |
| | IGF-1 | 396550 | 22758 | 266 | 1.03 (0.94 - 1.12) | 0.535 |
| | TSH | 11 | 8 | 8 | 0.97 (0.78 - 1.12) | 0.800 |
| **Male** | Estrogen | 236 | 4 | 1 | 1.33 (0.76 - 2.35) | 0.333 |
| | Testosterone | 27658 | 14176 | 156 | 1.01 (0.91 - 1.13) | 0.858 |
| | IGF-1 | 41186 | 24641 | 268 | 1.03 (0.94 - 1.12) | 0.554 |
| | TSH | 13 | 8 | 8 | 1.00 (0.86 - 1.15) | 0.941 |

(c) Susceptibility

| Sex | Exposure | Significant | Matched | Used | OR (95% CI) | *p* |
|---|---|---|---|---|---|---|
| **Female** | Estrogen | 20 | 10 | 1 | 1.14 (0.72 - 1.83) | 0.571 |
| | Testosterone | 7909 | 4990 | 96 | 1.03 (0.97 - 1.11) | 0.271 |
| | IGF-1 | 396550 | 23903 | 266 | 0.99 (0.95 - 1.04) | 0.774 |
| | TSH | 11 | 8 | 8 | 0.89 (0.80 - 0.99) | 0.029 |
| **Male** | Estrogen | 236 | 4 | 1 | 0.86 (0.65 - 1.13) | 0.274 |
| | Testosterone | 27658 | 13836 | 157 | 0.99 (0.94 - 1.04) | 0.607 |
| | IGF-1 | 41186 | 24873 | 240 | 0.99 (0.95 - 1.04) | 0.724 |
| | TSH | 13 | 8 | 8 | 0.97 (0.90 - 1.04) | 0.401 |
